# Application of ensemble machine learning algorithms on lifestyle factors and wearables for cardiovascular risk prediction

**DOI:** 10.1038/s41598-021-04649-y

**Published:** 2022-01-20

**Authors:** Weiting Huang, Tan Wei Ying, Woon Loong Calvin Chin, Lohendran Baskaran, Ong Eng Hock Marcus, Khung Keong Yeo, Ng See Kiong

**Affiliations:** 1 National Heart Centre Singapore, 5 Hospital Drive, Singapore, 169609 Singapore; 2 Institute of Data Science, National University of Singapore, Singapore, Singapore; 3 Singapore General Hospital, Singapore, Singapore

**Keywords:** Computational biology and bioinformatics, Cardiology, Health care, Medical research, Risk factors

## Abstract

This study looked at novel data sources for cardiovascular risk prediction including detailed lifestyle questionnaire and continuous blood pressure monitoring, using ensemble machine learning algorithms (MLAs). The reference conventional risk score compared against was the Framingham Risk Score (FRS). The outcome variables were low or high risk based on calcium score 0 or calcium score 100 and above. Ensemble MLAs were built based on naive bayes, random forest and support vector classifier for low risk and generalized linear regression, support vector regressor and stochastic gradient descent regressor for high risk categories. MLAs were trained on 600 Southeast Asians aged 21 to 69 years free of cardiovascular disease. All MLAs outperformed the FRS for low and high-risk categories. MLA based on lifestyle questionnaire only achieved AUC of 0.715 (95% CI 0.681, 0.750) and 0.710 (95% CI 0.653, 0.766) for low and high risk respectively. Combining all groups of risk factors (lifestyle survey questionnaires, clinical blood tests, 24-h ambulatory blood pressure and heart rate monitoring) along with feature selection, prediction of low and high CVD risk groups were further enhanced to 0.791 (95% CI 0.759, 0.822) and 0.790 (95% CI 0.745, 0.836). Besides conventional predictors, self-reported physical activity, average daily heart rate, awake blood pressure variability and percentage time in diastolic hypertension were important contributors to CVD risk classification.

## Introduction

Prevention of cardiovascular disease is based on the tenet that atherosclerotic disease occurs over time; risk factors and lifestyle are contributory, and appropriate modification can delay the onset of cardiovascular events. Previously established cardiovascular risk assessment models such as Framingham Risk Score (FRS)^1^, Systematic Coronary Risk Evaluation (SCORE)^2^ and QRISK2 score^3^ predict future risk based on well-established medical risk factors and pay little attention to lifestyle factors.

Recent studies highlighted the potential of lifestyle data in predicting cardiovascular risk^4^. The INTERHEART study found that nine risk factors including smoking, history of hypertension or diabetes, waist/hip ratio, dietary patterns, physical activity, consumption of alcohol, blood apolipoproteins (Apo), and psychosocial factors, accounted for 90% of the population attributable risk for myocardial infarction in men and 94% in women. These suggest room to improve cardiovascular risk assessment by incorporating new factors such as physical activity status, lifestyle and dietary habits^5,6^ alongside traditional risk predictors.

Prior studies have shown demonstrated increased cardiovascular risk from elevated blood pressure^7–9^; the PAMELA study found that combining office, rest and ambulatory blood pressure help predict cardiovascular mortality up to an area under curve of 0.81. Risk modelling have also been done of dietary and lifestyle behaviour, although the frequency of the studies is lower due to the need to administer detailed, time consuming food frequency questionnaires^10^. However due to the diverse data sources, and data types including time series, an integrated assessment tool combining lifestyle, diet, ambulatory physiological parameters, and clinical risk markers have not been performed to our knowledge.

Cardiovascular risk scores derived from traditional biostatistical methods such as logistic regression and Cox proportional hazard models^11–13^ provide parsimonious interpretation. However, their strict assumptions such as homoscedasticity, distribution normality and relationship linearity tend to oversimplify complex relationships and limit applications^14^. Machine learning algorithms (MLA) in studies^15–17^ were able to overcome these statistical drawbacks and improve discriminatory performance over traditional models. More recently, ensemble modelling techniques have also gained popularity such as in prediction of heart disease^18,19^, diabetes and hypertension^20^ cancer diagnosis and classification^21,22^. An ensemble model combines the different MLAs into one predictive model. Compared to a single model, an ensemble model is more robust and offers higher goodness-of-fit and better prediction accuracy^23^.

The aim of this paper is to investigate the additive value of four groups of risk factors, based on ease of information availability and regular clinical workflow, (lifestyle survey questionnaires, clinical blood tests, 24 h ambulatory blood pressure and heart rate monitoring) using ensemble MLA, in cardiovascular risk prediction. Due to limitations of the traditional biostatistical models, we used an ensemble MLA technique to learn the complex and non-linear interactions amongst the different groups of risk factors. To date, the application of ensemble MLA on lifestyle factors and clinical variables for individualised CVD risk assessment remains underexplored.

## Methods

### Data source and study population

Data used in this study was drawn from a SingHEART prospective longitudinal cohort study (ClinicalTrials.gov Identifier: NCT02791152). The study is a multi-ethnic population-based study conducted on healthy Asians, aged 21–69 years old without known diabetes mellitus or prior cardiovascular disease (Ischemic heart disease, stroke, peripheral vascular disease). The study complied with the Declaration of Helsinki and written informed consent were given by participants. The study was approved by the SingHealth Centralized Institutional Review Board.

We included 600 volunteers, aged of 30 years with valid calcium score, into the main analysis of this study. Two hundred volunteers under the age of 30 years, who did not have a calcium score were excluded, as the calcium score was the main outcome of our analysis.

Subset analysis for activity tracker data was performed on 430 out of the 600 volunteers who had adequate data. Although subjects recruited were issued an activity tracker to be worn over a period of five days with first and last days of the study being partial days, there was inconsistent wearing of the activity. Discounting the partial days, each subject would yield an activity log for three complete tracking days (or equivalent to days with > 20 valid hours of steps and sleep data)^24,25^. For data consistency and quality, subjects with improper activity tracker usage i.e. activity reading log less than five days and/or sleep reading log less than three days were censored.

### Markers of CVD risk and outcome

Coronary artery calcium (CAC) scoring was used as the modelling outcome. The coronary calcium is a specific marker of coronary atherosclerosis, a precursor for coronary artery disease^26^; it also reflects arterial age under the influence of underlying comorbidities and lifestyle. The CAC score was also regarded as the best marker for risk prediction of cardiovascular events^27,28^.

This study stratified subjects into two classes of CVD risk. Low risk if their coronary artery calcium score were 0, and high risk if calcium score were 100 and above. Subjects who did not fall into these 2 categories were considered intermediate risk.

The aim of this study is to look at how accurate the machine learning algorithm is in handling different data types, in the task of predicting high risk and low risk patients, based on calcium score.

### Data variables used for MLA: lifestyle survey questionnaires, clinical blood tests, ambulatory blood pressure and activity tracking data

Table 1 summarizes the data from SingHEART that was used in this study.

**Table 1:** List of risk factors used for prediction in this study.

**Survey Questionnaire** (Count: 30)
**Demographics:** Age gender, body mass index, race, single systolic blood pressure, single diastolic blood pressure, smoking history, waist and hip circumference **Medications:** consumption of medication for BP and dyslipidemia (i.e. diuretics, ACE inhibitors, Calcium antagonists, HMG CoA reductase inhibitors) **Social-demographics:** marital status, income, education, occupation, **Dietary preference:** Cups of coffee, fruits serving, vegetable serving, alcohol consumption **Sleep quality:** Sleep hours, sleep quality **Perception:** Stress level, lifestyle active **Therapy:** use of traditional/complementary medicines (i.e. traditional Chinese/Malay/Indian medicine, herbal remedies, acupuncture, chiropractic, vitamins, relaxation therapies, magnetic therapies, tai chi) **Medical history:** diabetes mellitus, hypertension, hyperlidemia, heart attack, heart failure, other heart disease **Family history:** ischemic heart disease
**24 h ambulatory blood pressure and heart rate** (Count: 17)
**Average overall readings:** systolic blood pressure, diastolic blood pressure, pulse rate, mean arterial pressure, heart rate **% Time ≥ Threshold for (H24, Awake, Nocturnal) readings:** systolic blood pressure, diastolic blood pressure **Average real variability readings for (H24, Awake, Nocturnal):** systolic blood pressure, diastolic blood pressure
**Clinical blood variables** (Count: 12)
ALT, AST, Albumin, CholesterolHDL, CholesterolLDL, CholesterolTotal, Creatinine, Glucose, Haemoglobin, Triglycerides, Urea, WBCCount
**Physical activity and sleep trackers** (Count: 16)
Calories burned, Steps, Distance, Floors, Minutes sedentary, Minutes lightly active, Minutes fairly active, Minutes very active, Activity calories, Minutes asleep, Minutes awake, Number of awakenings, Time in bed Minutes REM sleep, Minutes light sleep, Minutes deep sleep

Data variables were categorized into four groups; lifestyle survey questionnaires, blood test data, 24-h ambulatory blood pressure, and activity tracking data by commercially available Fitbit Charge HR^29^.

Data pre-processing, transformation and imputation were performed on the raw data. Variables selected were based on their a priori knowledge from previous publications on cardiovascular risk assessment^1–3^, and physician expert advice. In total, there were 30, 17, 12 and 16 unique variables in the respective groups: survey questionnaire, 24 h blood pressure and heart rate monitoring, blood tests and Fitbit data.

### Framingham risk score (FRS) as the comparator

The Framingham 10-year risk score was computed using seven traditional risk factors: gender, age, single timepoint systolic blood pressure, Total Cholesterol (TC), High Density Lipoprotein (HDL), smoking status and presence of diabetes. A Framingham risk score of < 10% is consider low risk, while ≥ 20% is considered high risk^30^.

### Modelling pipeline

**Figure 1:**
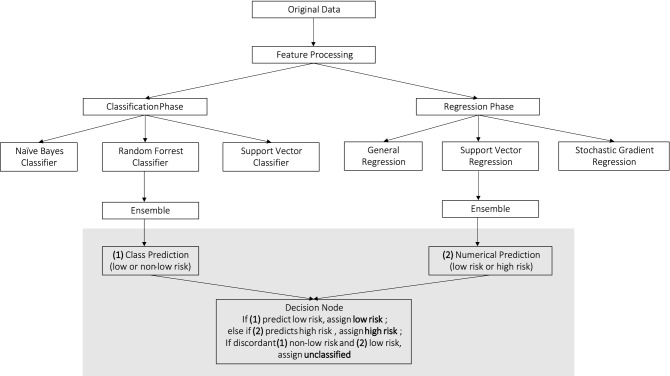
Modelling flow chart using ensemble MLA for cardiovascular risk prediction.

Figure 1 shows the methodological framework of the present study. Exploratory analysis showed that ensemble MLA classifiers were superior at discriminating low risk individuals while ensemble MLA regressors performed better identifying individuals with high CVD risk. To leverage on the merits of both the classifiers and regressors MLA, we used both approaches for our model.

The ensemble classifiers produce a binary prediction outcome; low or non-low risk. The ensemble regressors makes a numerical prediction on the calcium score for individuals classified as non-low risk, and stratify into three bins of low, high, and intermediate risk. The predicted numerical values may range from negative to positive number. Negative predicted values were first converted to zero and subsequently the continuous predictions were converted to discrete bins using unique value percentile discretization ensuring records with the same numerical prediction are assigned the same risk category. Finally, the prediction outcome resides in a decision node build on a rule-based logic. The decision node assigns an outcome of low risk if classifiers predict an individual to be low in CVD risk, high risk if classifier predicts non-low risk and regressor predicts high risk. Patients with incongruent classifiers and regressor outcomes are considered unclassified.

The ensemble models in both classification and regression phase each fit three base learners (naive bayes (NB), random forest (RF) and support vector classifier (SVC) for classification prediction, and generalized linear regression (GLM), support vector regressor (SVR) and stochastic gradient descent (SGD) for regression prediction). These base learners were chosen based on preliminary analysis, where these models showed efficiency in handling missing values and outliers.

The ensemble model then uses majority vote to determine the class label in classification phase. For the regression phase, the ensemble model averages the normalized predictions from the base regressor models to form a numerical outcome.

All models were trained on a stratified five-fold cross-validation. As SingHEART data had an imbalanced CVD risk distribution of risk based on the calcium score (low risk 63.4%, high risk 8.3%, intermediate risk 18.7%) we oversampled the training set for the minority class labels to allow model to better learn features from the under-represented classes^31^. The data were first partitioned into five mutually exclusive subsets, with each subset sharing the same proportion of class label as original dataset. At each iteration, the MLAs trained on four parts (80%) and validated on the fifth, the holdout set (20%). The process repeats five times, with five different but overlapping training sets. The resulting metrics from each fold were averaged to produce a single estimate.

To simulate access to the different variable groups as per clinical workflow and ease of information availability, we assessed the performance of individual variable group, and in combination as per the following:

Model 1: Survey Questionnaire.

Model 2: 24 h ambulatory blood pressure and heart rate.

Model 3: Clinical blood results.

Model 4: Model 1 + Model 2.

Model 5: Model 1 + Model 3.

Model 6: Model 1 to Model 3.

Model 6*: Model 1 to Model 3 with feature selection.

Model 7: Physical activity and sleep trackers (exploratory subset analysis).

Variables in model 6* were reduced using SVC recursive feature elimination with cross-validation (SVC-RFECV) method to automatically select the best set of predictors that yield the highest area under Receiver Operating Characteristic curves (AUC). Model 1–6 were trained using 600 subjects.

We also performed exploratory analysis using MLA on the Fitbit Charge HR data (Model 7). Model 7 was trained on a subset of 430 subjects constrained by availability of valid activity tracking data.

### Evaluation methodology and metrics

Since no single metric can objectively evaluate the cardiovascular risk prediction, we evaluate the performance of our models at CVD risk class level using a panel of metrics; sensitivity, specificity, positive predictive value (PPV), negative predictive value (NPV), F1-score and Area under Receiver Operating Characteristic curves (AUC). Overall discriminative ability of the model was described by the area under received operating characteristic curve (ROC). All AUC metrics were accompanied by 95% confidence interval (CI) and standard deviation (SD).

To better understand the relative importance of different risk factors, we conduct a post-hoc approach to rank the variables by their contribution to CVD risk prediction. Feature importance were obtained from the SVC algorithm where the relative importance was determined by the absolute size of the coefficients in relation to others. All statistical analyses were conducted on Python version 3.7 environment and all MLAs and evaluation metrics were constructed using Scikit-learn libraries.

## Results

### Baseline characteristics

The SingHEART data consist of 800 anonymized individuals. After excluding cases no coronary calcium scan and other missing information, 600 subjects were used for this analysis. Tables 2, 3, 4, 5 presents the demographics, lifestyle survey questionnaires, clinical blood test and activity tracking data characteristics stratified by CVD risk class. The p-values displayed are obtained by comparing low and high risk categories. Continuous variables are presented in mean values with ± standard deviations while variables are categorical responses are expressed in count and percentage.

**Table 2:** Demographics by risk categories.

Risk factors	Total (n = 600)	Low risk (Agatston = 0) (n = 421)	High risk (Agatston > = 100) (n = 55)	Intermediate risk (Agatston 1–99) (n = 124)	*P-Values
Age	49.6 ± 9.2	47.02 ± 8.68	58.55 ± 6.55	54.39 ± 7.34	0.0000
Gender (Male 1, Female 0)	276 (46)%	155 (36.82)%	43 (78.18)%	78 (62.9)%	0.0001
Body Mass Index (BMI)	23.63 ± 3.72	23.59 ± 3.71	23.85 ± 3.63	23.7 ± 3.82	0.0000
Waist circumference	83.09 ± 11.01	82.38 ± 11.11	85.95 ± 9.71	84.24 ± 11	0.0000
Hip circumference	95.15 ± 9.82	95.37 ± 9.26	93.27 ± 14.54	95.21 ± 9.07	0.0000
**Race**					
Chinese	561 (93.5)%	396 (94.06)%	51 (92.73)%	114 (91.94)%	0.0000
Indian	18 (3)%	10 (2.38)%	3 (5.45)%	5 (4.03)%	0.0000
Malay	10 (1.67)%	7 (1.66)%	1 (1.82)%	2 (1.61)%	0.0001
Others	11 (1.83)%	8 (1.9)%	0 (0)%	3 (2.42)%	0.9993
**Income**					
< $3000	235 (39.17)%	150 (35.63)%	25 (45.45)%	60 (48.39)%	0.5008
≥ $3000—$4999	146 (24.33)%	116 (27.55)%	14 (25.45)%	16 (12.9)%	0.0005
≥ $5000	219 (36.5)%	155 (36.82)%	16 (29.09)%	48 (38.71)%	0.0027
**Occupation**					
Not working	115 (19.17)%	73 (17.34)%	14 (25.45)%	28 (22.58)%	0.0005
Blue-collar worker	32 (5.33)%	20 (4.75)%	4 (7.27)%	8 (6.45)%	0.0000
Pink-collar worker	45 (7.5)%	32 (7.6)%	2 (3.64)%	11 (8.87)%	0.0000
White-collar worker	404 (67.33)%	293 (69.6)%	35 (63.64)%	76 (61.29)%	0.0459
Other workers	4 (0.67)%	3 (0.71)%	0 (0)%	1 (0.81)%	0.9993
Marital status (Married 1, else 0)	473 (78.83)%	327 (77.67)%	52 (94.55)%	94 (75.81)%	0.0000
Highest education (at least university degree 1, else 0)	310 (51.67)%	225 (53.44)%	27 (49.09)%	58 (46.77)%	0.8927
Smoking history	48 (8)%	31 (7.36)%	8 (14.55)%	9 (7.26)%	0.0000
Alcohol consumption	59 (9.83)%	44 (10.45)%	6 (10.91)%	9 (7.26)%	0.0000
**Medical history**					
Personal/family history of Diabetes Mellitus	201 (33.5)%	135 (32.07)%	15 (27.27)%	51 (41.13)%	0.0012
Personal/family history of Hyperlipidemia	110 (18.33)%	74 (17.58)%	9 (16.36)%	27 (21.77)%	0.0000
Personal/family history of Hypertension	275 (45.83)%	191 (45.37)%	22 (40)%	62 (50)%	0.1407
Personal/family history of ischemic heart disease	69 (11.5)%	44 (10.45)%	8 (14.55)%	17 (13.71)%	0.0000
Medication for BP and dyslipidemia	12 (2)%	2 (0.48)%	4 (7.27)%	6 (4.84)%	0.0000

**Table 3:** Self-reported lifestyle factors and 24 h blood pressure and heart rate monitoring data by risk categories.

Lifestyle factors	Total (n = 600)	Low risk (Agatston = 0) (n = 421)	High risk (Agatston > = 100) (n = 55)	Intermediate risk (Agatston 1–99) (n = 124)	*P-values
Coffee (number of cups per day)	0.98 ± 1.02	0.98 ± 1.03	1.07 ± 1.03	0.93 ± 0.97	0.0000
Fruits (servings per day)	1.32 ± 0.86	1.3 ± 0.9	1.31 ± 0.6	1.38 ± 0.83	0.0000
Vegetables (servings per day)	1.93 ± 0.95	1.94 ± 0.99	1.87 ± 0.77	1.9 ± 0.9	0.0000
Sleep Hours	6.57 ± 1.03	6.55 ± 1.03	6.4 ± 0.87	6.71 ± 1.07	0.0000
**Sleep quality**					
Bad	7 (1.17)%	7 (1.66)%	0 (0)%	0 (0)%	0.9993
Fairly bad	53 (8.83)%	32 (7.6)%	9 (16.36)%	12 (9.68)%	0.0000
Fairly good	397 (66.17)%	281 (66.75)%	33 (60)%	83 (66.94)%	0.1407
Very good	138 (23)%	98 (23.28)%	13 (23.64)%	27 (21.77)%	0.0002
Stress Level	4.46 ± 2.14	4.6 ± 2.15	4.47 ± 2	3.98 ± 2.12	0.0000
Lifestyle Active	5.55 ± 2.26	5.29 ± 2.28	6.25 ± 1.99	6.11 ± 2.13	0.0000
Traditional medicine, Therapies and Vitamins	268 (44.67)%	187 (44.42)%	30 (54.55)%	51 (41.13)%	0.5008
**24 Hours blood pressure monitoring**					
Systolic BP single reading	128.1 ± 17.25	124.91 ± 16.43	137.8 ± 13.14	134.61 ± 18.22	0.0000
Diastolic BP single reading	78.19 ± 12.92	76.21 ± 12.82	84.09 ± 11.14	82.26 ± 12.31	0.0000
Average daily systolic BP	116.59 ± 13.27	113.99 ± 12.29	125.09 ± 11.94	121.63 ± 14.1	0.0000
Average daily diastolic BP	73.93 ± 8.72	72.3 ± 7.94	79.18 ± 8.59	77.14 ± 9.54	0.0000
Average daily mean aterial pressure (MAP)	88.09 ± 9.36	86.24 ± 8.52	93.96 ± 8.73	91.73 ± 10.23	0.0000
Average daily pulse pressure (PP)	42.57 ± 7.3	41.57 ± 7.09	46 ± 5.79	44.46 ± 7.83	0.0000
Average daily heart rate (HR)	71.47 ± 8.59	71.33 ± 8.71	73.51 ± 8.39	71.03 ± 8.19	0.0000
% time awake systolic BP ≥ 135	0.18 ± 0.25	0.14 ± 0.22	0.34 ± 0.27	0.27 ± 0.3	0.0000
% time awake diastolic BP ≥ 85	0.23 ± 0.26	0.19 ± 0.23	0.37 ± 0.3	0.32 ± 0.31	0.0000
% time nocturnal systolic BP ≥ 120	0.23 ± 0.29	0.19 ± 0.27	0.42 ± 0.34	0.3 ± 0.32	0.0000
% time nocturnal diastolic BP ≥ 70	0.42 ± 0.32	0.37 ± 0.31	0.61 ± 0.31	0.52 ± 0.33	0.0000
% time average daily systolic BP ≥ 120	0.4 ± 0.31	0.34 ± 0.29	0.62 ± 0.26	0.5 ± 0.31	0.0000
% time average daily diastolic BP ≥ 80	0.31 ± 0.27	0.27 ± 0.24	0.47 ± 0.28	0.4 ± 0.31	0.0000
Awake systolic BP ARV	9.04 ± 2.12	8.66 ± 1.88	10.32 ± 2.23	9.75 ± 2.42	0.0000
Nocturnal systolic BP ARV	8.97 ± 3.11	8.77 ± 3.16	9.61 ± 3.28	9.35 ± 2.79	0.0000
Awake diastolic BP ARV	43.39 ± 7.48	42.32 ± 7.15	47.04 ± 5.96	45.43 ± 8.25	0.0000
Nocturnal diastolic BP ARV	40.92 ± 7.16	40.25 ± 6.98	43.43 ± 6.97	42.06 ± 7.51	0.0000
Average daily systolic BP ARV	8.87 ± 1.93	8.53 ± 1.78	10.03 ± 1.94	9.49 ± 2.09	0.0000
Average daily diastolic BP ARV	42.64 ± 7.14	41.67 ± 6.88	45.98 ± 5.79	44.46 ± 7.76	0.0000

**Table 4:** Blood test variables by risk categories.

Blood tests	Total (n = 600)	Low risk (Agatston = 0) (n = 421)	High risk (Agatston > = 100) (n = 55)	Intermediate risk (Agatston 1–99) (n = 124)	*P-Values
Alanine aminotransferase (ALT)	21.38 ± 13.02	20.09 ± 12.05	28.13 ± 19.49	22.75 ± 11.59	0.0000
Aspartate transaminase (AST)	26.54 ± 8.33	25.57 ± 7.71	31.82 ± 12.43	27.5 ± 7.08	0.0000
Albumin	43.14 ± 2.36	42.96 ± 2.43	43.75 ± 2.15	43.45 ± 2.17	0.0000
Cholesterol high-density lipoprotein (HDL)	1.49 ± 0.34	1.5 ± 0.33	1.48 ± 0.35	1.47 ± 0.35	0.0000
Cholesterol low-density lipoprotein (LDL)	3.39 ± 0.83	3.29 ± 0.82	3.68 ± 0.83	3.6 ± 0.81	0.0000
Cholesterol total	5.43 ± 0.94	5.31 ± 0.91	5.8 ± 0.96	5.64 ± 0.94	0.0000
Creatinine	68.52 ± 15.72	66.39 ± 15.42	74.42 ± 16.49	73.13 ± 14.81	0.0000
Glucose	5.29 ± 0.69	5.21 ± 0.66	5.67 ± 1.05	5.41 ± 0.51	0.0000
Haemoglobin	13.64 ± 1.47	13.45 ± 1.5	14.26 ± 1.28	14.02 ± 1.31	0.0000
Triglycerides	1.18 ± 0.68	1.12 ± 0.65	1.42 ± 0.68	1.29 ± 0.73	0.0000
White blood cell count (WBC)	5.81 ± 1.6	5.86 ± 1.66	5.61 ± 1.27	5.74 ± 1.52	0.0000
Urea	4.45 ± 1.13	4.33 ± 1.1	4.63 ± 1.02	4.79 ± 1.19	0.0000

**Table 5:** Fitbit Charge HR data by risk categories.

Wearables	Total (n = 600)	Low risk (Agatston = 0) (n = 421)	High risk (Agatston > = 100) (n = 55)	Intermediate risk (Agatston 1–99) (n = 124)	*P-values
Calories burned	2161.41 ± 478.39	2102.27 ± 451.04	2447.03 ± 559.49	2213.68 ± 470.08	0.0000
Steps	9406.76 ± 3198.63	9207.3 ± 3170.53	10,274.93 ± 3326.31	9631.18 ± 3174.39	0.0000
Distance	6.52 ± 2.33	6.36 ± 2.34	7.18 ± 2.33	6.73 ± 2.24	0.0000
Floors	9.02 ± 7.44	8.99 ± 7.7	8.74 ± 6.75	9.26 ± 6.93	0.0000
Minutes sedentary	873.27 ± 120.03	881.41 ± 121.88	836.56 ± 112.48	864.75 ± 114.6	0.0000
Minutes lightly active	220.44 ± 64.78	219.02 ± 63.86	224.04 ± 69.9	223.37 ± 65.73	0.0000
Minutes fairly active	18.39 ± 17.48	15.62 ± 13.59	34.37 ± 27.21	19.53 ± 18.53	0.0000
Minutes very active	19.77 ± 19.56	17 ± 16.99	34.29 ± 28.56	21.67 ± 18.76	0.0000
Activity calories	943.78 ± 359.86	892.63 ± 337.24	1204.36 ± 448.75	982.18 ± 323.04	0.0000
Minutes asleep	447.4 ± 90.75	451.4 ± 90.18	452.61 ± 93.08	431.44 ± 90.7	0.0000
Minutes awake	37.19 ± 16.39	37.29 ± 15.95	35.6 ± 20.82	37.68 ± 15.42	0.0000
Number of awakenings	2.78 ± 2.91	2.77 ± 2.91	2.22 ± 1.31	3.12 ± 3.43	0.0001
Time in bed	485.65 ± 98.04	489.78 ± 96.68	489.23 ± 101.39	470.1 ± 100.36	0.0000
Minutes REM sleep	1.33 ± 7.52	1.17 ± 7.37	0.28 ± 1.85	2.42 ± 9.48	0.7914
Minutes light sleep	4.02 ± 21.58	3.41 ± 20.18	0.92 ± 6.14	7.61 ± 29.43	0.7492
Minutes deep sleep	0.92 ± 5.1	0.82 ± 5.05	0.14 ± 0.95	1.61 ± 6.33	0.8419

*Compares between low risk and high risk categories.

The cohort had a mean age of 49.6 years (range 29 to 69 years) and 46% were males. All the factors in the Framingham Risk score were significantly different between the low and high-risk classes on univariate analysis.

In novel parameters such as 24 h ambulatory blood pressure and heart rate, higher measures and derivatives of blood pressure measurement were congruously associated with increased risk (p-value < 0.001). Patients with lower risk had a lower mean average heart rate.

### Model performance

AUC for 4 individual variable groups of survey questionnaires, clinical blood tests, 24 h blood pressure and heart rate monitoring, and activity tracker all performed better than the conventional FRS for both low risk and high risk patients (p-value < 0.001). Of all the individual variable groups, survey questionnaires achieved the highest AUC score for both low risk (AUC 0.715 95% CI 0.681–0.750) and high risk (AUC 0.710 95% CI 0.653–0.766). Adding clinical blood tests to survey questionnaire did not improve AUC for both the low risk (p-value = 0.441) and high risk (p-value = 0.715) categories. Adding 24 h blood pressure and heart rate monitoring significantly improved the overall performance compared to the Model 1 using survey questionnaire only, with significant p-values of 0.01 for low risk and 0.005 for high risk groups.

Table 6 demonstrated the cross validated model performance, by evaluating sensitivity, specificity, positive predictive value, negative predictive value, F1 and AUC. FRS had high sensitivity (91.4%) and low specificity (32.9%) in detecting low risk individuals, and low sensitivity (3.7%) and high specificity (99.3%) in detecting high risk individuals. The MLA models achieved a better balance between sensitivity and specificity.

**Table 6:** Performance of conventional Framingham Risk Score and MLA models by variable groups in low risk categories.

Model 1: Survey questionnaire					
Model 2: 24 h ambulatory blood pressure and heart rate					
Model 3: Clinical blood results					
Model 4: Model 1 + Model 2					
Model 5: Model 1 + Model 3					
Model 6: Model 1 + Model 2 + Model 3					
Model 6*: Model 1 to Model 3 with feature selection					
Model 7: Physical activity and sleep trackers					

	FRS	Model 1	Model 2	Model 3	Model 4
**Low risk**					
Sensitivity (95% CI), SD	0.914 (0.891 0.936), 0.014	0.797 (0.765 0.829), 0.019	0.696 (0.66 0.732), 0.022	0.662 (0.622 0.699), 0.023	0.817 (0.783 0.848), 0.02
Specificity (95% CI), SD	0.329 (0.27 0.387), 0.036	0.633 (0.573 0.695), 0.037	0.559 (0.497 0.619), 0.037	0.577 (0.514 0.635), 0.036	0.609 (0.548 0.671), 0.037
Positive predictive value (95% CI), SD	0.762 (0.731 0.792), 0.018	0.836 (0.805 0.867), 0.018	0.788 (0.753 0.821), 0.021	0.787 (0.752 0.82), 0.021	0.831 (0.8 0.86), 0.018
Negative predictive value (95% CI), SD	0.62 (0.536 0.706), 0.05	0.57 (0.513 0.626), 0.034	0.438 (0.386 0.492), 0.032	0.421 (0.367 0.472), 0.031	0.585 (0.526 0.649), 0.037
F1 (95% CI), SD	0.831 (0.81 0.852), 0.013	0.816 (0.791 0.84), 0.015	0.739 (0.71 0.766), 0.017	0.719 (0.688 0.748), 0.018	0.824 (0.798 0.847), 0.015
AUC (95% CI), SD	0.622 (0.592 0.653), 0.019	0.715 (0.681 0.75), 0.021	0.627 (0.593 0.662), 0.022	0.62 (0.583 0.655), 0.022	0.713 (0.678 0.747), 0.021

	Model 5	Model 6	Model 6*	Model 7	
**Low risk**					
Sensitivity (95% CI), SD	0.793 (0.762 0.822), 0.019	0.814 (0.781 0.844), 0.019	0.821 (0.79 0.851), 0.019	0.764 (0.723 0.805), 0.025	
Specificity (95% CI), SD	0.666 (0.606 0.727), 0.037	0.615 (0.556 0.68), 0.037	0.761 (0.709 0.815), 0.032	0.634 (0.565 0.701), 0.041	
Positive predictive value (95% CI), SD	0.848 (0.817 0.879), 0.018	0.833 (0.802 0.862), 0.018	0.89 (0.863 0.915), 0.016	0.822 (0.784 0.858), 0.022	
Negative predictive value (95% CI), SD	0.577 (0.521 0.633), 0.034	0.583 (0.529 0.643), 0.035	0.643 (0.59 0.697), 0.033	0.549 (0.481 0.618), 0.042	
F1 (95% CI), SD	0.819 (0.794 0.843), 0.015	0.823 (0.799 0.848), 0.014	0.854 (0.831 0.875), 0.013	0.791 (0.76 0.82), 0.018	
AUC (95% CI), SD	0.729	0.714 (0.681 0.749), 0.021	0.791 (0.759 0.822), 0.019	0.699 (0.66 0.737), 0.024	
	(0.695 0.765), 0.021				

	FRS	Model 1	Model 2	Model 3	Model 4
High risk					
Sensitivity (95% CI), SD	0.037 (0 0.083), 0.025	0.637 (0.526 0.745), 0.065	0.526 (0.413 0.636), 0.068	0.472 (0.358 0.589), 0.07	0.654 (0.547 0.758), 0.063
Specificity (95% CI), SD	0.993 (0.987 0.998), 0.004	0.783 (0.755 0.812), 0.017	0.739 (0.708 0.77), 0.019	0.749 (0.718 0.778), 0.018	0.803 (0.776 0.83), 0.017
Positive predictive value (95% CI), SD	0.333 (0 0.714), 0.216	0.23 (0.177 0.289), 0.034	0.17 (0.126 0.217), 0.029	0.16 (0.112 0.208), 0.029	0.253 (0.194 0.311), 0.036
Negative predictive value (95% CI), SD	0.91 (0.892 0.929), 0.012	0.955 (0.939 0.97), 0.01	0.939 (0.919 0.957), 0.012	0.933 (0.912 0.952), 0.012	0.958 (0.943 0.972), 0.009
F1 (95% CI), SD	0.065 (0 0.145), 0.044	0.337 (0.268 0.41), 0.042	0.256 (0.194 0.317), 0.038	0.238 (0.173 0.3), 0.039	0.363 (0.29 0.432), 0.043
AUC (95% CI), SD	0.515 (0.496 0.538), 0.013	0.71 (0.653 0.766), 0.034	0.632 (0.574 0.691), 0.035	0.61 (0.554 0.672), 0.036	0.729 (0.674 0.783), 0.033

	Model 5	Model 6	Model 6*	Model 7	
**High risk**					
Sensitivity (95% CI), SD	0.655 (0.547 0.755), 0.063	0.726 (0.627 0.825), 0.06	0.82 (0.733 0.903), 0.053	0.776 (0.673 0.873), 0.061	
Specificity (95% CI), SD	0.787 (0.759 0.816), 0.017	0.805 (0.778 0.832), 0.017	0.761 (0.731 0.788), 0.018	0.776 (0.741 0.812), 0.021	
Positive predictive value (95% CI), SD	0.238 (0.186 0.296), 0.034	0.275 (0.217 0.335), 0.036	0.258 (0.208 0.315), 0.033	0.289	
				(0.229 0.355), 0.038	
Negative predictive value (95% CI), SD	0.957 (0.941 0.972), 0.009	0.967 (0.953 0.98), 0.008	0.977 (0.963 0.988), 0.007	0.967 (0.949 0.983), 0.01	
F1 (95% CI), SD	0.348 (0.281 0.421), 0.042	0.397 (0.328 0.468), 0.043	0.392 (0.329 0.459), 0.041	0.42 (0.348 0.492), 0.045	
AUC (95% CI), SD	0.721 (0.666 0.774), 0.033	0.766 (0.715 0.816), 0.031	0.79 (0.745 0.836), 0.028	0.776 (0.722 0.826), 0.032	

**Figure 2:**
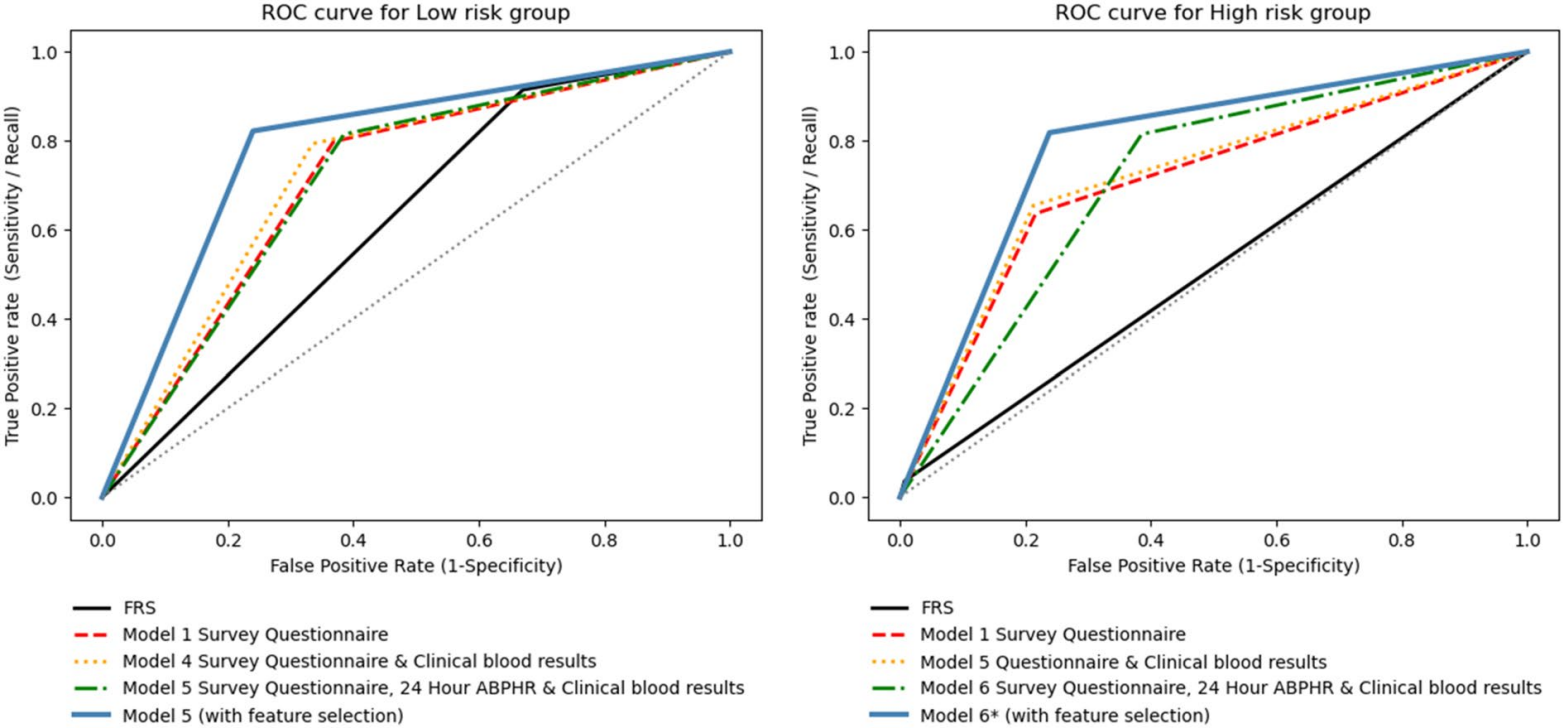
ROC curves for low risk group (left) and high risk group (right). Colours and line style represent the prediction performance for different models. Prediction performance for both low and high risk groups were significantly better in model 5* compared to FRS.

The continuous net reclassification of the lifestyle questionnaire survey variables over FRS in our population were 18% for low cardiovascular risk prediction and 39% for high cardiovascular risk prediction. For the combined Model 6*, the continuous net reclassification over FRS were 25% and 119% for low and high risk categories respectively. Figure 2 shows the receiver operating curves comparing the various models in the low and high cardiovascular risk groups based on their CAC.

Conventional risk factor variables such as age, blood pressure readings, gender and family history of ischemic heart disease were the top ranking contributors to risk prediction in Model 1 (lifestyle survey). Other less conventional but important contributors include self-assessed physical activity and sleep hours.

For Model 2, 24-h blood pressure and heart rate monitoring, percentage time of blood pressure > 120/80 mmHg appeared to be most important compared to other blood pressure readings. Average real variability of blood pressure during wake period and percentage time of nocturnal diastolic hypertension ≥ 70 mmHg were also featured by the model.

In Model 3, clinical blood test variables, conventional risk factor variables of glucose, AST, haemoglobin, albumin and cholesterol readings topped the feature importance ranking.

In the exploratory analysis concerning activity tracking data, minutes in “fairly active” and “very active”, and sleep-related activity log particularly, minutes of REM and minutes of light sleep data were more important features than average daily steps, distance and floors.

**Figure 3:**
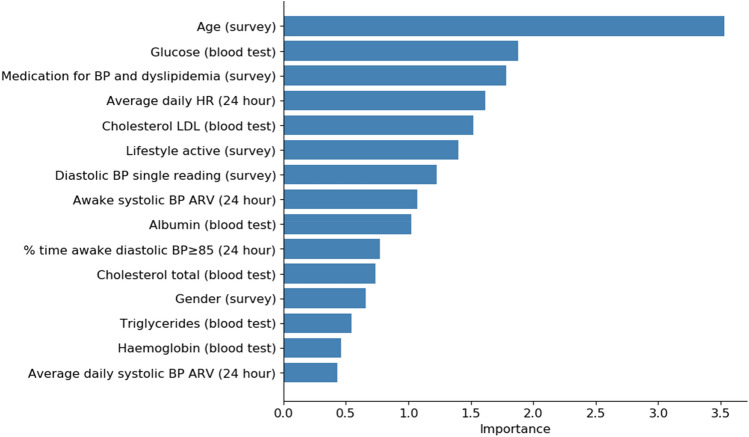
The top 15 features of MLA models showing the relative importance of the different variables in CVD risk prediction. Age, glucose, cholesterol LDL, wake period blood pressure variability, medication for BP and dyslipidemia, triglycerides and albumin reading were some common predictors across the different versions.

Summing all the factors, age, medication for blood pressure and dyslipidemia, albumin, glucose, wake period diastolic hypertension, LDL cholesterol, self-reported physical activity were the top predictors across multiple models (see Fig. 3).

## Discussion

This study looked at four groups of variables (survey questionnaires, clinical blood tests, 24 h ambulatory blood pressure and heart rate monitoring and activity trackers) and their association with CAC score, for cardiovascular risk classification. We designed our modelling approach by first examining the discriminatory performance of variables in readily accessible, self-reported survey questionnaire group, which did not require equipment or blood test. The incremental contribution to the models’ performance were examined by sequentially adding other groups of variables, simulating availability of information as per clinical workflow. This was compared to the traditional FRS framework.

Previous well-established risk scores such as FRS^1^, SCORE^2^ and QRISK2 score^3^ were mostly derived using traditional risk factors like age, total cholesterol, HDL, systolic BP, smoking and diabetes, excluding physical activity, lifestyle and dietary habits. In our study, we found the risk estimation derived from the FRS framework to be suboptimal with an AUC of 0.622 and 0.515 when applied on the Asian population of low and high risk profiles respectively. The moderate performance of FRS in our cohort corresponds prior published literature in primary care clinics in Asia^32^, although some other larger cohort studies suggest higher areas under the curve of up to 0.768^33,34^. While traditional risk factors remain robust, we hypothesize that non-traditional, personalized risk factors such as dietary intake, physical activity and ambulatory blood pressure can contribute to individual cardiovascular risk assessment. Recent studies such as CARDIA^35^ has demonstrated such potential, and we explored these novel variables using machine learning algorithms. Beyond enhancing individualised cardiovascular risk prediction, this allows users to identify modifiable behavioural factors that can improve risk profiles.

In this healthy Asian ethic population, we found that variables from survey questionnaire achieved an AUC of 0.715 and 0.710 for individuals with low and high CVD risk respectively. Interestingly, we observed that the addition of clinical blood tests on top of survey questionnaire risk factors did not significantly enhance ensemble MLA’s ability in classifying low and high cardiovascular risk, with non-significant p-values when the combined model (Model 5) was compared to the survey questionnaire model (Model 1). This suggests that potential of designing MLA-based survey questionnaire that can be easily implemented, for risk stratification. The survey questionnaire, without need for blood tests is less cumbersome and can be implemented as a population-wide survey, to risk stratify patients. This finding complements the currently available health risk appraisals^36^ which highlights health risk, but does not diagnose or risk stratify patients, which our current model can do. Our model can potentially vary risk outputs based on changes in lifestyle behaviours included within the questionnaires; this gives patients an actionable plan beyond medications, to reduce their cardiovascular risk.

The ideal cut-off for hypertension has been a constant debate^37–39^ and our study revealed interesting predictors which requires further study. While in-clinic and self-measured blood pressure are single timepoint measurements, they do not reflect the actual variability and time-in-range for blood pressure when a person is performing their activities. There has been varying results in the correlation of blood pressure with cardiovascular events and end-organ outcomes^40–42^. However there has been supporting studies, suggesting that the blood pressure of 120/80 will be optimal in preventing adverse cardiovascular events, especially strokes^42–44^. Our MLA models have identified that a greater percentage time in blood pressure < 120/80 is associated with a better cardiovascular profile. This brings about a new concept of time in range, which is an increasingly important measure in diabetology^45^, Our study suggests that time-in-range may be extrapolated to hypertension. Additionally, our study also indicated the importance of the daytime variability of blood pressure, which is increasingly recognised to be a marker of cardiovascular risk to be also an important contributor. This concept is supported by recent studies demonstrating association of increased variability with cardiovascular risk^46–48^. Although current blood pressure monitoring devices are single time-point, future wearables may be able to provide the time-in-range readouts and diurnal variability, which were important components associated with atherosclerosis in our study.

The physical activity data in our subgroup also revealed interesting findings in that active minutes were more important than total step count in predicting coronary atherosclerosis. This suggests that achieving the required metabolic equivalents and target heart rate is more important than distance travelled or steps taken in line with physical activity guideline of achieving 150 min of moderate physical exercise per week^49^.

A practical application of our findings would be in terms of statin prescription, by being able to modestly discern low risk and non-low risk, defined as calcium score 0 and calcium more than 0. The American College of Cardiology suggests patients with zero calcium score on coronary arteries (very low risk patients) can defer of statin therapy in the absence of elevated cardiac risk of ≥ 20% in 10 years^50^. In this study, we found our ensemble MLA performed better than the Framingham risk score in identifying low risk individuals (p-value < 0.001).

While there have been numerous studies on CVD risk prediction, studies involving the application of ensemble MLA on contemporary risk factors such as lifestyle and ambulatory physiological data on Asian population remains understudied. In^51^, a study modelled on survey-based responses suggest promising findings in detection of cardiovascular risk patients. Our work extends previous findings by examining the predictive value of the different groups of risk factors and their combined effect to classify CVD risk among healthy asymptomatic individuals in Asian population. Another key contribution of our study is identifying novel risk factors which contributes to CVD risk classification. Our approach prioritizes on easily obtainable variables where inputs to the risk prediction models is not restricted to laboratory or other advanced cardiac imaging test for classification of CVD risk; our models are versatile in that while providing more information helps refine risk prediction, simple health behaviour and lifestyle inputs can already provide a risk prediction. From a population health perspective, this helps create patient self-awareness of health status, and motivate higher risk patients to seek therapy early, thereby lowering health care expenditure in long run. This work therefore present opportunities for use of self-assessed questionnaire data as a preliminary low-cost option to screen healthy individuals for CVD risk. Finally, we also demonstrated the suitability of machine learned models when on applied on dataset with numerous potential predictors. The use of an ensemble modelling technique to synthesize the outcome of multiple base learners can increase model’s robustness and prevent overfitting.

## Limitation and future work

In our subanalysis of physical activity Fitbit charge HR parameters, we found that data from such devices were unable to risk stratify patients with high confidence. We attribute the inconclusive performance due to relatively small sample size of patients with adequate Fitbit data, especially for patients in the high risk categories. Patients with high CVD risk accounts for 9.2% (55 out of 600) of the dataset in comparison to 70.2% (421) patients in low risk. Congruent with prior studies, we found associations between activity tracker determined physical activities, sleeping hours and sleep quality with cardiovascular health^52^, but we will need a larger sample size study before such parameters can be reliably incorporated into a risk model.

Our study is limited by a smaller sample size of patient with high CVD risk defined as calcium score ≥ 100. Individuals with high CVD risk accounts for 20.1% (124) of the dataset in comparison to 70.2% (421) individuals in low risk. We addressed the class-imbalance problem with synthetic minority oversampling technique (SMOTE) by generating synthetic samples of the minority class. SMOTE will not only mitigates the problem of overfitting caused by random oversampling, it will also create more instances of the minority class for MLA to learn^53^. We also performed only internal validation. This model is built on data from an Asian population, applicability to other populations will require further calibration. Additionally, we only assessed the performance of the model in high and low risk patients; this is due to the limited sample size and to prevent overfitting of the data. We will present this data after the completion of our prospective trial consisting of at least 2000 patients.

As an extension to current work, longitudinal follow-up information will be added enriched the dataset by examining the continuity of each variable across different time points. A prospective trial evaluating this model is planned to provide a larger sample size for learning and model evaluation. Deep learning frameworks capable of capturing the complex interactions while preserving the order and temporal elements of the multiple readings can be explored in place of MLAs for more accurate CVD risk classification.

## Data Availability

The datasets that support the findings of this study are not publicly available due to personal data protection and ethical reasons. The data can be made available and the corresponding authors may be contacted for access to data for an IRB approved collaboration.

## Abbreviations

ALT: Alanine aminotransferase
AST: Aspartate transaminase
AUC: Area under receiver operating characteristics
ARV: Average real variability
BP: Blood pressure
CAC: Coronary artery calcium
CVD: Cardiovascular disease
FPR: False positive rates
FRS: Framingham risk score
HDL: High-density lipoprotein cholesterol
LDL: Low-density lipoprotein cholesterol
MLA: Machine learning algorithms
NHCS: National Heart Centre Singapore
NPV: Negative predictive values
PPV: Positive predictive values
ROC: Receiver operating characteristics
SMOTE: Synthetic minority oversampling technique
TPR: True positive rates
